# Pooled effector library screening in protoplasts rapidly identifies novel *Avr* genes

**DOI:** 10.1038/s41477-024-01641-y

**Published:** 2024-02-26

**Authors:** Taj Arndell, Jian Chen, Jana Sperschneider, Narayana M. Upadhyaya, Cheryl Blundell, Nathalie Niesner, Megan A. Outram, Aihua Wang, Steve Swain, Ming Luo, Michael A. Ayliffe, Melania Figueroa, Thomas Vanhercke, Peter N. Dodds

**Affiliations:** 1 CSIRO Agriculture and Food, Canberra, Australian Capital Territory Australia; 2 Present Address: Centre for Agriculture and the Bioeconomy, Queensland University of Technology, Brisbane, Queensland Australia

**Keywords:** Effectors in plant pathology, High-throughput screening, Microbe

## Abstract

Crop breeding for durable disease resistance is challenging due to the rapid evolution of pathogen virulence. While progress in resistance (*R*) gene cloning and stacking has accelerated in recent years^1–3^, the identification of corresponding avirulence (*Avr*) genes in many pathogens is hampered by the lack of high-throughput screening options. To address this technology gap, we developed a platform for pooled library screening in plant protoplasts to allow rapid identification of interacting *R*–*Avr* pairs. We validated this platform by isolating known and novel *Avr* genes from wheat stem rust (*Puccinia graminis* f. sp. *tritici*) after screening a designed library of putative effectors against individual *R* genes. Rapid *Avr* gene identification provides molecular tools to understand and track pathogen virulence evolution via genotype surveillance, which in turn will lead to optimized *R* gene stacking and deployment strategies. This platform should be broadly applicable to many crop pathogens and could potentially be adapted for screening genes involved in other protoplast-selectable traits.

## Abstract (web summary)

The authors developed a platform for rapid identification of interacting plant immune receptors and pathogen avirulence proteins by library screening in protoplasts, then used it to identify new wheat stem rust *Avr* genes recognized by known wheat resistance genes.

## Main

Crop pathogens greatly reduce agricultural productivity and are a persistent threat to global food security^4,5^. The most effective and sustainable approach to mitigate crop disease is through breeding of resistance (*R*) genes into crop varieties. Most *R* genes encode immune receptors, such as nucleotide-binding leucine-rich repeat (NLR) proteins, that directly or indirectly recognize pathogen effectors, known as avirulence (Avr) proteins^6,7^. These immune recognition events induce plant defence responses, often including localized cell death, which limit pathogen spread. However, pathogens continuously evolve to escape recognition via mutation of *Avr* genes. Thus, understanding *Avr* gene diversity in pathogen populations is critical for the effective deployment of *R* genes in breeding or by gene stacking.

Rust fungi (order Pucciniales) cause serious diseases in many crop plants, especially among staple cereal crops including wheat, barley, oat and corn^8^. For example, wheat stem rust disease is caused by the fungus *Puccinia graminis* f. sp. *tritici* (*Pgt*), and newly arisen virulent strains such as Ug99 have caused devastating losses and threaten global wheat production^9,10^. Rust fungi have large genomes (for example, from ~150 Mbp to over 1.0 Gbp^8^) with two separate haploid nuclei and encoding thousands of potential effectors. Although hundreds of rust resistance loci are described in cereals (many no longer effective), only three corresponding *Avr* genes (*AvrSr27*, *AvrSr35*, *AvrSr50*)^11–13^ have been identified in *Pgt* and only two from other cereal rusts (*AvrRppC* and *AvrRppK* from southern corn rust *P. polysora*^14,15^).

**Fig. 1:**
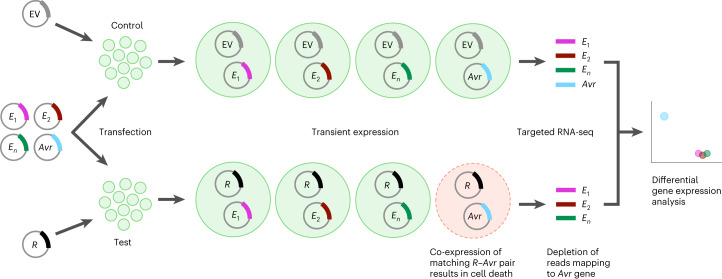
Schematic of pooled effector library screening process to identify interacting *R*–*Avr* pairs. Protoplast populations are co-transformed with a pooled library comprising hundreds of effector genes (*E*_1_, *E*_2_, *E*_*n*_, *Avr*) from a pathogen along with either a known *R* gene or an empty vector (EV) for the negative control. The library MOT, defined as the number of plasmid molecules per cell (for each library construct), is chosen such that each library construct is expressed in an independent subpopulation of cells. Each cell individually receives a random but limited number of different constructs from the library together with either the empty vector or the *R* gene. In the presence of the *R* gene, protoplasts that express a matching *Avr* effector gene undergo cell death, while cells expressing the same *Avr* effector gene in the negative control remain alive. Living protoplasts are subsequently collected from both transformed populations and subjected to targeted (library-specific) RNA-seq. The expression of each effector in the library is then compared between the two samples (differential gene expression analysis). *Avr* gene candidates are identified by their decreased expression in the sample expressing the corresponding *R* gene.

Approaches to functionally test *Avr* gene candidates usually involve pairwise transient co-expression of individual candidate *R*–*Avr* gene combinations by polyethylene glycol (PEG)-mediated protoplast transformation or leaf agroinfiltration to detect induced cell death^16,17^. However, these methods assay candidate effectors one-by-one, so screening many effectors requires highly labour-intensive and time-consuming sequential assays^13,14^. The increasing availability of high-quality pathogen genome sequences combined with recent advances in fungal and oomycete effector prediction from sequence or structural features^18,19^ presents an opportunity to design and synthesize effector libraries to systematically screen genome-wide pathogen effector complements for immuno-recognition. We therefore set out to develop a platform for pooled effector library screening in plant protoplasts to enable rapid identification of interacting *R*–*Avr* pairs. Figure 1 outlines the screening scheme in which an *R* gene of interest and a pooled effector gene library are co-delivered to protoplasts such that a subpopulation of cells expressing the *Avr* gene undergoes cell death, resulting in the depletion of *Avr* gene transcripts in the living cell population. Library-specific RNA-seq and differential gene expression analysis is then used to identify effectors showing reduced expression when co-expressed with specific *R* genes relative to the empty vector control.

**Fig. 2:**
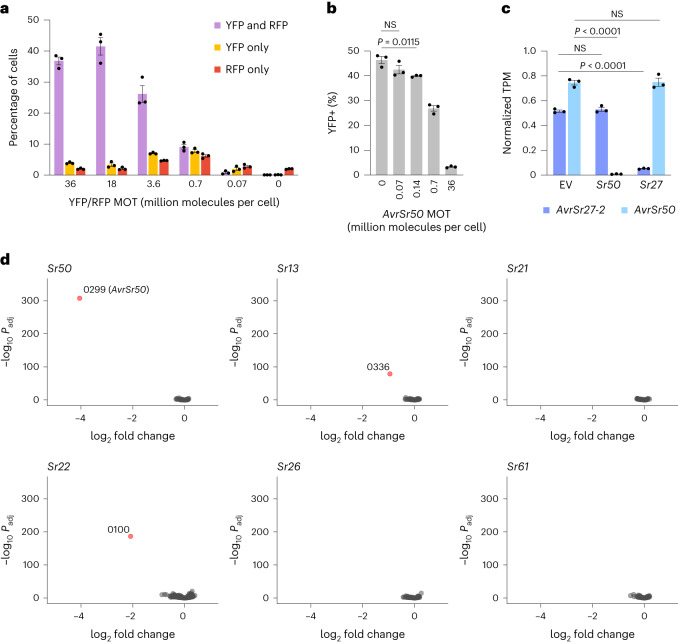
Optimization and validation of pooled effector library screening for rapid identification of interacting *R*–*Avr* pairs. **a**, Wheat protoplasts were transformed with YFP and RFP reporter constructs at various MOTs (million plasmid molecules per cell) along with an empty vector whose amount was varied such that the combined MOT of the three constructs remained constant at 72 million plasmid molecules per cell. The percentage of cells showing fluorescence in the YFP, RFP or both wavelengths was determined by flow cytometry. Mean ± s.e.m. of 3 biological replicates. **b**, Wheat protoplasts were co-transformed with YFP, *Sr50* and one of a series of mock libraries comprising *AvrSr50* at various MOTs within a background of *AvrSr35* (combined MOT of 36 million plasmid molecules per cell for the two constructs). Plot shows the percentage of YFP-positive living cells determined by flow cytometry. Mean ± s.e.m. of 3 biological replicates (dots), with relevant significant differences indicated (NS, not significant; two-tailed unpaired *t*-test assuming equal variances). **c**, Expression levels of *AvrSr27-2* and *AvrSr50* in a mock effector library screen. Wheat protoplasts were co-transformed with a mock library consisting of *AvrSr50* (MOT of 0.14 million plasmid molecules per cell) and *AvrSr27-2* (MOT of 0.14 million plasmid molecules per cell) within a background of *AvrSr35* (MOT of 100 million plasmid molecules per cell) and either *Sr50*, *Sr27* or an empty vector (MOT of 36 million plasmid molecules per cell). Relative expression levels of *AvrSr27-2* and *AvrSr50* were determined by targeted RNA-seq (shown in transcripts per million (TPM), normalized to *AvrSr35* expression). Mean ± s.e.m. of 3 replicates, with significant differences indicated (two-tailed unpaired *t*-test assuming equal variances). **d**, Differential gene expression analysis of a pooled stem rust effector library comprising 696 predicted effectors co-transformed into wheat protoplasts with the *R* genes *Sr50*, *Sr13c*, *Sr21*, *Sr22*, *Sr26* or *Sr61* compared to the empty vector. Graphs show volcano plots of differential expression (*x* axis) versus *P*_adj_ (*y* axis) for each effector construct (dots). Effector gene constructs showing significantly reduced expression (red dots) within each treatment are labelled with their library ID number.

A key requirement for this pooled library screening process is that different library constructs are expressed in independent cell subpopulations at a sufficient level to induce cell death and allow for differential screening. However, it has been suggested that library screening in protoplasts may not be possible, since large amounts of plasmid are typically used to ensure sufficient expression^20^. While reducing the plasmid concentration should reduce the frequency of co-transformation, this may also reduce the level of transgene expression in transformed cells and lower effector expression may not be sufficient for NLR-mediated cell death activation. We therefore first addressed these potential limitations by defining plasmid to cell ratios at which independent expression of constructs is favoured. We express these ratios as the multiplicity of transfection (MOT), which is calculated as the number of plasmid molecules present per protoplast cell in a transformation reaction. For example, in a transformation with 10 μg of a 5.4 kbp plasmid and 50,000 protoplasts, the MOT is 36 million molecules per cell. We used flow cytometry to test for independent transformation of protoplasts with two reporter gene constructs encoding either yellow or red fluorescent protein (YFP, RFP) under the control of the maize ubiquitin 1 promoter (Ubi1p). The two reporter constructs and an empty vector were co-delivered to protoplasts as a series of mock libraries in which reporter construct MOTs ranged from 0.07 to 36 million molecules per cell and the amount of empty vector was varied such that the combined MOT of the three constructs remained constant at 72 million molecules per cell (Fig. 2a and Supplementary Fig. 1). At high MOTs (36, 18, 3.6 million molecules per cell) a high proportion of protoplasts expressed both fluorescent markers, with only a small fraction expressing a single marker. However, the proportion of doubly transformed cells dropped at lower MOTs. At 0.7 million molecules per cell, ~7% of cells expressed YFP or RFP alone, while 9% expressed both. Only ~1% of cells expressed both markers when delivered at 0.07 million molecules per cell, while a larger proportion expressed a single marker (2–3% for each). This indicated that differential library screening may be feasible when individual constructs are delivered in the range of 0.07–0.7 million molecules per cell. However, at these lower MOTs we observed reduced fluorescence levels in individual transformed cells compared with the high MOT transformations (Supplementary Fig. 1), suggesting that each cell was transformed with fewer copies of the constructs, leading to lower expression. Therefore, we also examined whether transformation with an *Avr* gene at these low MOTs could lead to induction of cell death in a subpopulation of cells as required for library screening. Previous assays for cell death in protoplasts have relied on high expression of corresponding *R* and *Avr* genes along with a luciferase reporter gene, such that *Avr* recognition resulted in extensive cell death and greatly reduced luciferase activity in the cell suspension as a whole relative to a control^3,17,21^. However, this assay would not discriminate whether a slight reduction in reporter activity at low *Avr* gene MOTs is due to a small proportion of cells undergoing cell death and not producing luciferase or to a larger proportion of cells with reduced expression. Therefore, we modified this approach to develop an individual cell scoring assay to quantify the proportion of cells responding with cell death in a protoplast suspension. This also eliminates some sources of variation in the assay since the output is normalized to the number of living cells and is independent of initial cell number and absolute reporter gene expression. In this individual cell assay, a fluorescent protein reporter (YFP) is used instead of luciferase and transformed protoplasts are analysed individually for fluorescence by flow cytometry. Co-expression of three known wheat stem rust *R*–*Avr* pairs, *Sr50*-*AvrSr50*, *Sr27*-*AvrSr27-2* and *Sr35*-*AvrSr35* (all under the control of Ubi1p), resulted in a significant decrease in the proportion of YFP-positive protoplasts in the living (propidium iodide-negative) population of cells, compared with the controls where single *R* or *Avr* genes or non-matching *R–Avr* pairs were expressed (Supplementary Fig. 2). This assay allows for quantification of the proportion of cells showing cell death in a protoplast suspension, as opposed to total expression of the reporter in the population as a whole. To determine whether cell death responses could still be recorded at low MOTs, we delivered *AvrSr50* at various MOTs along with *Sr50*. To maintain a constant total amount of DNA in each transformation and simulate library screening conditions, we also included *AvrSr35* to give a combined MOT of 36 million molecules per cell for the *AvrSr50* and *AvrSr35* constructs together. We observed a small but significant reduction in the proportion of YFP-positive cells with *AvrSr50* at an MOT of 0.14 million molecules per cell (Fig. 2b), indicating that a subpopulation of cells expressed *AvrSr50* and underwent cell death at this plasmid concentration. The proportion of YFP-positive cells was reduced by close to half at an MOT of 0.7 million molecules per cell, suggesting that too many cells were undergoing cell death to allow for efficient library selection at this plasmid concentration. No significant reduction in the proportion of YFP-positive cells was observed when *AvrSr50* was delivered at an MOT of 0.07 million molecules per cell, although it is possible that selectable cell death still occurs at this or lower MOTs but is not detectable against the variation in transformation. We therefore selected an MOT of 0.14 million molecules per cell as appropriate for library screening. This represents a compromise between the frequency of independent transformation, cell death response levels, and library size and complexity, allowing ~700 constructs to be delivered at a total MOT of 100 million molecules per cell, which is sufficiently diverse to support comprehensive screening.

As a final validation step for the library screening process, we tested whether differential *Avr* gene expression could be used to identify interacting *R*–*Avr* pairs. For this, we transformed protoplasts with a mock library comprising *AvrSr50* and *AvrSr27-2*, each delivered at an MOT of 0.14 million molecules per cell and representing individual clones in an effector library, as well as *AvrSr35* delivered at an MOT of 100 million molecules per cell and representing the remainder of the library. The mock library was co-transformed with *Sr50*, *Sr27* or an empty vector (all at an MOT of 36 million molecules per cell). RNA-seq analysis of *Avr* gene expression in protoplasts at 24 h post transformation showed that *AvrSr27-2* and *AvrSr50* were both expressed when the mock library was co-transformed with an empty vector (Fig. 2c). However, expression of *AvrSr50* was substantially reduced when co-expressed with *Sr50* but not with *Sr27*, and *AvrSr27-2* was reduced when co-expressed with *Sr27* but not with *Sr50*. Thus, the effects of each *Avr* gene could be independently assessed by their relative expression when co-delivered under simulated library screening conditions, suggesting that differential expression could be used to identify *Avr* gene candidates from an effector library.

Having established appropriate experimental conditions for library screening, we synthesized expression constructs for 696 predicted *Pgt* effectors selected from the Pgt21-0 reference genome annotation^10^ as genes encoding secreted proteins of less than ~330 amino acids and with expression patterns similar to known *Avr* genes^11^. These plasmids were pooled in equimolar amounts and co-transformed at an MOT of 0.14 million molecules per cell for each individual construct into protoplasts with either an empty vector or one of seven separate *R* gene constructs encoding *Sr50* (ref. ^22^), *Sr27* (ref. ^11^), *Sr13c* (ref. ^23^), *Sr21* (ref. ^24^), *Sr22* (ref. ^1^), *Sr26* (ref. ^25^) or *Sr61* (ref. ^25^) (each at an MOT of 36 million molecules per cell). Pgt21-0 is avirulent on wheat lines expressing these resistance genes^11,13,25,26^. Library-specific RNA-seq and differential gene expression analysis was used to identify effectors showing reduced expression when co-expressed with specific *R* genes relative to the empty vector control. Two independent screens correctly identified *AvrSr50* as a single gene showing significantly reduced expression in the presence of *Sr50* only (Fig. 2d and Supplementary Fig 3). Likewise, the five known variants of *AvrSr27* (refs. ^11,27^) all showed significantly reduced expression only when co-expressed with *Sr27* (Supplementary Fig. 3). Thus, this platform can specifically identify single and multiple *Avr* genes from a complex library of effector candidates screened against different *R* genes in parallel. Two effectors, with library IDs 0336 and 0100, showed significantly reduced expression only in the presence of *Sr13c* or *Sr22*, respectively, and represent candidates for *AvrSr13* and *AvrSr22*. Both candidates were identified in the two independent screens, highlighting the robustness of the platform. No effectors showed reduced expression in the presence of *Sr21*, *Sr26* or *Sr61*. This may be because these *Avr* genes were not present in the library. For instance, *AvrSr35* was not included in the library due to its unusually large size (~550 aa), which did not pass the filtering criteria for synthesis. The synthesized library represents about half of the annotated genes encoding secreted proteins expressed in haustoria, but the reference annotation may not include all *Avr* genes (see below). Thus, identification of *Avr* gene candidates from four out of seven *R* genes screened (two out of five *R* genes whose matching *Avr* was unknown) represents a high detection rate and synthesis of a larger effector library based on improved annotation^28^ may allow identification of additional candidates. Alternatively, these *Avr* genes may not have been identified because they require a higher expression level threshold for induction of cell death or due to lack of NLR protein expression.

**Fig. 3:**
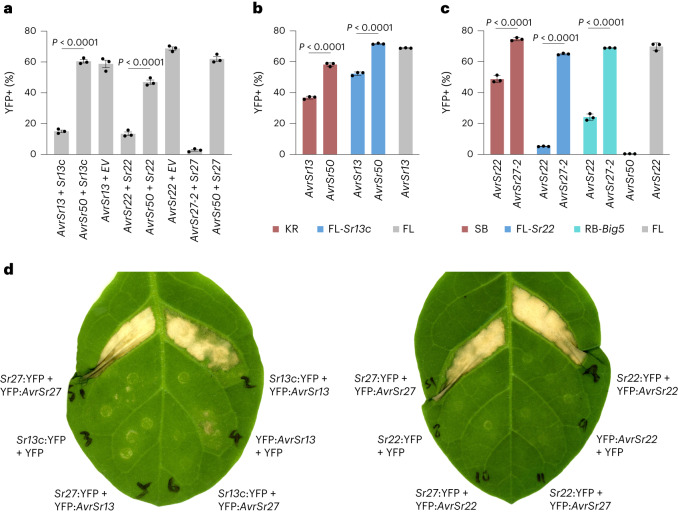
Validation of *AvrSr13* and *AvrSr22* candidates. **a**, Wheat protoplasts (cv. Fielder) were co-transformed with YFP, an *Avr* gene (*AvrSr13*, *AvrSr22*, *AvrSr27-2* or *AvrSr50*) and an *R* gene (*Sr13c*, *Sr22* or *Sr27*) or empty vector. **b**, YFP was co-transformed with *AvrSr13* or *AvrSr50* into protoplasts derived from wheat lines Kronos (KR, containing native *Sr13a*), Fielder (FL) or transgenic FL containing the *Sr13c* transgene (FL-*Sr13c*). **c**, The YFP reporter was co-transformed with *AvrSr22* or *AvrSr27*-*2* into protoplasts derived from wheat lines Schomburgk (SB, containing native *Sr22*), FL, transgenic FL containing the *Sr22* transgene (FL-*Sr22*), or transgenic Robin containing a five-*R-*gene cassette including *Sr22* (RB-*Big5*)^3^. Plots in **a**, **b** and **c** show the percentage of YFP-positive living cells determined by flow cytometry. Mean ± s.e.m. of 3 replicates, with significant differences indicated for relevant pairwise comparisons (two-tailed unpaired *t*-test assuming equal variances). All gene constructs were delivered at an MOT of 36 million plasmid molecules per cell. **d**, *Agrobacterium*-mediated transient co-expression of Sr27, Sr13c or Sr22 proteins (C-terminally fused to YFP) with AvrSr27-2, AvrSr13 or AvrSr22 (N-terminally fused to YFP) or YFP alone in *N. tabacum* leaves. Agrobacterial cultures were delivered at OD_600_ of 0.4 (*R* gene constructs) or 0.7 (*Avr* gene constructs).

Specific recognition of the *AvrSr13* and *AvrSr22* candidates by *Sr13c* and *Sr22*, respectively, was confirmed by co-transformation of protoplasts with the individual *Avr* gene candidates and the corresponding *R* genes, which resulted in a significant reduction in YFP-positive cells compared with the *R* or *Avr* genes alone or non-matching *R*–*Avr* gene pairs (Fig. 3a). Similarly, specific recognition was also observed following transformation of the individual *Avr* gene candidates into protoplasts derived from stable transgenic wheat lines expressing either *Sr13c* or *Sr22*, as well as protoplasts derived from wheat cultivars expressing endogenous *Sr13a* or *Sr22* genes (cv. Kronos and cv. Schomburgk, respectively) (Fig. 3b,c). Similarly, agroinfiltration assays in *N. tabacum* and *N. benthamiana* also showed cell death induction upon transient co-expression of *AvrSr13* with *Sr13c* or *AvrSr22* with *Sr22*, but not with the *R* or *Avr* genes alone or non-matching *R*–*Avr* gene pairs (Fig. 3d and Supplementary Fig. 4).

AvrSr13 is encoded by PGT21_021053 on chromosome 1A in the Pgt21-0 genome reference, with a null allele on chromosome 1B indicating that this strain is heterozygous for avirulence to *Sr13*. However, the Ug99 *Pgt* isolate (race TTKSK) is homozygous for identical *AvrSr13* genes (PGTUg99_007363 and unannotated) in the A and C haplotypes and is therefore probably homozygous for avirulence on *Sr13*. AvrSr22 is encoded by PGT21_017626 on chromosome 16B in the Pgt21-0 genome reference, and the alternative allele on chromosome 16A in Pgt21-0 contains a related sequence that is not annotated but encodes a mature protein with nine amino acid differences from AvrSr22. Mapping RNA-seq data from Pgt21-0 haustoria and infected plant samples^29^ confirmed the expression of this transcript during infection at similar levels to *AvrSr22*, suggesting that it is a functional gene which we designated as the *AvrSr22-2* allele. The Ug99 genome contains sequences identical to both *AvrSr22* (PGTUg99_032354) and *AvrSr22-2* (tig00002160, unannotated). Co-expression of *AvrSr22-2* with *Sr22* in wheat protoplasts resulted in a significant decrease in the proportion of YFP-positive cells similar to that seen with *AvrSr22*, but expression of *AvrSr22-2* alone had no effect (Supplementary Fig. 5a). Similarly, co-expression of *AvrSr22-2* with *Sr22*, but not with YFP, also induced cell death in agroinfiltrated *N. tabacum* and *N. benthamiana* leaves (Supplementary Fig. 5b). Since AvrSr22 and AvrSr22-2 are both recognized by Sr22, Pgt21-0 and Ug99 are probably homozygous for avirulence on *Sr22*. The observation that the Ug99 strain is homozygous for avirulence on *Sr13c* and *Sr22* suggests that these resistance genes are more likely to provide durable resistance to Ug99-derived *Pgt* strains than *Sr27*, *Sr35* or *Sr50* for which this strain is heterozygous for avirulence^10,11,13^.

In summary, we developed a library screening platform to rapidly identify interacting pairs of plant immunoreceptors and pathogen Avr effectors that confer disease resistance. The library complexity and concentration conditions used here were chosen on the basis of evidence of co-transformation frequency and cell death responses for specific control genes at different MOTs and proved successful for isolation of several *Avr* genes. However, optimal conditions may vary depending on various factors including vector size, promoter/terminator activity, *R*–*Avr* combination, plant species and protoplast transformation efficiency. An important question is ‘what is the maximum library size that can be screened in this way?’ Given that protoplast transformation is well established in numerous plant species^30^, this platform could be applied to accelerate advances in many critical plant diseases. This will provide molecular tools needed for surveillance of genetic variation at *Avr* loci in pathogen populations and enable the creation of *R–Avr* gene atlases^31^ to inform breeding and deployment of disease-resistant crops. For instance, the homozygosity of the Ug99 stem rust strain for *Avr* genes corresponding to *Sr13c* and *Sr22* provides a rationale for prioritizing these resistance genes in breeding programs and *R* gene stacking approaches^3^ targeting durable resistance to this race group. In addition, newly cloned *Avr* genes can be used to confirm the functionality of corresponding *R* genes in transgenic stack lines to expedite the development of next-generation disease-resistant crops (as demonstrated here with *AvrSr22* used to confirm the functionality of *Sr22* in our transgenic line RB-*Big5*). Extending the platform to identify *Avr* genes corresponding to unknown *R* genes in resistant types (including non-hosts) will be an important next step towards realizing the full potential of this new approach. Finally, given the feasibility of direct library screening in protoplasts, there is potential to devise other cell screening protocols to identify genes controlling other important biological traits.

## Methods

### Vector design and construction

pTA22-YFP (Supplementary Fig. 6) is a high-copy (pUC19 origin of replication) plasmid containing the coding sequence for Venus yellow fluorescent protein (YFP) flanked by the maize ubiquitin 1 promoter (including the first intron) and a 35S:NOS double terminator. The negative control empty vector pTA22 was created by digestion of pTA22-YFP with *Kpn*I-*Sac*I, followed by blunting and self-ligation to remove the YFP coding sequence. The destination vectors pTA22-GW and pTA22-GW-PBS were created by replacing the YFP coding sequence in pTA22-YFP with a synthesized (GeneArt) Gateway cassette (1,741 bp and 1,761 bp, respectively) via restriction/ligation (*Kpn*I-*Sac*I). pTA22-GW-PBS is identical to pTA22-GW except that pTA22-GW-PBS also contains a 20-bp reverse primer binding site (PBS) for the primer FS_cDNA_R (5′-TGCTAGATCTCGACAGTACG-3′) between the Gateway cassette and the 35S:NOS double terminator.

The coding sequence of the wheat stem rust resistance gene *Sr50* and the open reading frames including introns of the wheat stem rust resistance genes *Sr27*, *Sr35*, *Sr13c*, *Sr21*, *Sr22*, *Sr26* and *Sr61* were cloned into pTA22-GW via Gateway LR reaction (Invitrogen) to create the expression vectors pTA22-Sr50, pTA22-Sr27, pTA22-Sr35, pTA22-Sr13c, pTA22-Sr21, pTA22-Sr22, pTA22-Sr26 and pTA22-Sr61, respectively. The open reading frames in the *R* gene expression vectors were sequence verified. *Avr* gene open reading frames (*AvrSr50*, *AvrSr27-2*, *AvrSr35*, *AvrSr22-2* and *avrSr50-B6*) were cloned into pTA22-GW-PBS via Gateway LR reaction to create the expression vectors pTA22-AvrSr50-PBS, pTA22-AvrSr27-2-PBS, pTA22-AvrSr35-PBS, pTA22-AvrSr22-2-PBS and pTA22-avrSr50-B6-PBS, respectively, with the insertions validated by digestion with diagnostic restriction enzymes chosen to cut within both the vector backbone and insert sequences.

For transient expression in *Nicotiana* spp., the coding sequences of AvrSr13 and AvrSr22 were transferred from the library constructs pTA22-0336-PBS and pTA22-0100-PBS, respectively, into the pDONR207 vector via Gateway BP reaction (Invitrogen). The predicted coding sequences of *Sr13c*, *Sr22* and *AvrSr22-2* were synthesized and subcloned into pDONR207 via Gateway BP cloning. *R* and *Avr* gene sequences were then transferred into the binary vectors pAM-35s-GWY-YFPv and pBIN19-35s-YFPv-GWY, respectively, by Gateway LR reaction as previously described^21,32^. Plasmids used in this study are described in Extended Data Table 1.

### Effector library design and construction

Effector candidates from *Pgt* were selected from the genome reference annotation for Pgt21-0 (ref. ^10^) (NCBI BioProject PRJNA516922) and a differential expression analysis of all genes encoding secreted proteins that identified eight clusters of genes with different expression profiles during the *Pgt* infection cycle^11^. Candidates for inclusion in the *Pgt* effector library were selected on the basis of the following filtering criteria: present in secreted protein gene expression clusters 2, 3 or 7; length <1,000 nt after removal of the signal peptide encoding region; expression level in haustoria >5 transcripts per million (TPM); SignalP3.0 signal peptide prediction probability >0.5; unique translated protein sequence in candidate set. The coding sequences of the resulting 718 putative effectors were codon optimized for wheat using GeneOptimizer. Additional sequences designed to facilitate synthesis and cloning were added immediately upstream (5′-AGGCTTCACC-3′) and immediately downstream (5′-CCATACCCAGCTTTCTTGTACAAAGTGGTTTGATCGTACTGTCGAGATCTAGCA*ACGCGATCGGAGGCGCTCATTATACGCAGATTCTTTATCGAAGCTGAGGGTGTGCCCGCTGTAACCCGCAAAGCCGTCAATATACAATCCTGACCAAATAGGAGACTGAACCGGTTTGGTAGCAGATAAGTTGCTTGGTGCCG*-3′) of the optimized coding sequences. It was a manufacturing requirement that all synthesized fragments be at least 300 bp long. Therefore, the minimum length of randomly generated filler sequence (italicized above in the downstream sequence) was used where necessary (the 94 smallest effectors) to meet this minimum length. Of the 718 putative effectors, 696 were successfully synthesized and cloned (Twist Bioscience) into pTA22-GW-PBS to create a library of expression vectors with names pTA22-0001-PBS to pTA22-0718-PBS (Supplementary Table 1).

### Effector library pooling and propagation

The 696 effector library constructs were individually resuspended in 100 µl IDTE buffer (10 mM Tris, 0.1 mM EDTA, pH 7.5; IDTE, 11-05-01-05) and then pooled in equimolar amounts (150 fmol per construct) using a JANUS G3 automated liquid handling workstation. The final concentration of the pooled library was 132 ng µl^−1^ as measured using Nanodrop and 61 ng µl^−1^ as measured using Qubit 4. To propagate the pooled library, 2.5 µl of pooled library DNA was transformed into 50 µl of ElectroMAX Stbl4 competent *Escherichia coli* cells (Invitrogen, 11635018) by electroporation (BioRad MicroPulser, Ec2 setting [2.5 kV, ~5 s]), followed by addition of 2.5 ml pre-warmed SOC medium (included with the competent cells). Outgrowth without selection proceeded at 37 °C for 1 h with shaking at 220 rpm. After outgrowth, cells were spun down and resuspended in 2.5 ml of Luria-Bertani (LB) medium. The culture was then diluted 1 in 200 using LB medium and 300 µl of the diluted culture was spread on a 14.5-cm-diameter LB agar plate containing 50 µg ml^−1^ carbenicillin. Colonies were scraped off the plates using a cell spreader and LB medium, and then transferred to Falcon tubes. Cells were spun down, supernatant was removed and the pellets were then stored at −20 °C before plasmid DNA isolation.

### Plasmid DNA isolation

pTA22-YFP was isolated using the MACHEREY NAGEL NucleoBond Xtra Maxi Plus EF kit (740426.50). All other plasmids were isolated using the MACHEREY NAGEL NucleoBond Xtra Midi Plus EF kit (740422.50). The pooled library was isolated using the QIAGEN EndoFree Plasmid Giga kit (12391). Isolated plasmid DNA was resuspended at a concentration of 1 µg μl^−1^ (measured on NanoDrop).

### Protoplast isolation and transformation

Wheat (*Triticum aestivum*) seeds were planted in 13 cm pots (12 seeds per pot) containing Martins Seed Raising and Cutting Mix supplemented with 3 g l^−1^ osmocote. Seedlings were grown in a growth cabinet at 24 °C on a cycle of 12 h light (~100 µmol m^−2^ s^−1^) and 12 h dark for 7–8 days. The cultivar Fielder was used for library screening and experiments involving co-transformation of *R* genes with *Avr* genes or candidates. The cultivars Kronos (native *Sr13a*), Fielder transgenic containing *Sr13c* (FL-*Sr13c*), Schomburgk (native *Sr22*), Fielder transgenic containing *Sr22* (FL-*Sr22*) and Robin transgenic containing a five-*R*-gene cassette including *Sr22* (RB-*Big5*) were also used for further validation of *Avr* candidates. Protoplast isolation and transformation was carried out as described previously^33^, with minor modifications. Following enzymatic digestion of cell walls, released mesophyll protoplasts were filtered through a 40 μm nylon cell strainer. Protoplasts were then centrifuged for 3 min at 80 *g*, resuspended in W5 solution (2 mM MES-KOH pH 5.7, 5 mM KCl, 125 mM CaCl_2_, 154 mM NaCl) and incubated on ice. The settled protoplast pellet was resuspended in an appropriate volume of MMG solution (4 mM MES-KOH pH 5.7, 0.4 M mannitol, 15 mM MgCl_2_). The protoplast concentration was determined by cell counting on a haemocytometer and subsequently adjusted to 2.5 × 10^5^ cells per ml using MMG solution. NEBioCalculator was used to calculate the number of plasmid molecules used in each transformation, according to the following formula: moles dsDNA (mol) = mass of dsDNA (g)/((length of dsDNA (bp) × 615.96 g mol^−1^ bp^−1^) + 36.04 g mol^−1^). For example, 10 μg of a 5.4 kbp plasmid corresponds to 3.0 pmol (=0.00001g/((5,400 bp × 615.96 g mol^−1^ bp^−1^) + 36.04 g mol^−1^)) or 1.8 × 10^12^ DNA molecules (=3.0 × 10^−12^ moles dsDNA × 6.022 × 10^23^ molecules mol^−1^). For standard individual transformations involving single *R–Avr* gene combinations, 3 pmol of each vector was mixed with 200 μl of protoplasts (50,000 protoplasts; MOT = 36 million molecules per cell) and ~230 μl of PEG solution (40% w/v PEG-4000, 0.2 M mannitol, 100 mM CaCl_2_) in a 2 ml tube. The DNA/protoplast/PEG mixture was homogenized by gently flicking the tube, and then incubated for 15–30 min at room temperature before adding 940 μl of W5 solution and gently inverting the tube to mix and stop the transformation reaction. Transformed protoplasts were centrifuged for 2 min at 100 *g*, resuspended in 650 μl W5 solution, transferred to 12-well cell culture plates and incubated at 23 °C for 24 h in the dark. For mock library screening, transformation reactions were scaled up 11×. For pooled library screening, transformation reactions were scaled up 5× or 8× (for example, 8× transformation reactions contained 296–387 μl vector DNA (pooled library + *R* gene or empty vector), 1.6 ml of protoplasts and 1.95 ml PEG solution; stopped with 8 ml W5 solution) in 25 ml tubes and transformed protoplasts were incubated in cell culture flasks. Transformations were performed in triplicate for all treatments and controls.

### Flow cytometry

After the 24 h incubation period, protoplasts were transferred to 2 ml tubes, stained with propidium iodide (10 µg ml^−1^) and then subjected to flow cytometry using the Invitrogen Attune NxT flow cytometer to detect fluorescence properties of individual cells. Propidium iodide fluorescence (excitation 561 nm, emission filter 620/15 nm) was measured as an indicator for living protoplasts (no fluorescence) and YFP fluorescence (excitation 488 nm, emission filter 530/30 nm) was measured as an indicator of the reporter gene expression. Gates were set to delimit the protoplast population within all events, living cells within the protoplast population and YFP-positive cells within the living population. The percentage of YFP-positive protoplasts in the living (propidium iodide-negative) population was used to assess the transformation efficiency and quantify R–Avr induced cell death. When RFP was used as a reporter, cells were not stained with propidium iodide and RFP fluorescence was detected using an excitation of 561 nm and an emission filter of 620/15 nm. Attune NxT Software 3.1 was used for data collection and analysis. Two-tailed unpaired *t*-tests assuming equal variances were performed using Prism 10.0.1 software.

### Messenger (m)RNA extraction and complementary (c)DNA synthesis/PCR

After the 24 h incubation period, protoplast samples (three replicates of each treatment and control) were transferred to individual 5 ml tubes and centrifuged for 3 min at 150 *g*. The supernatant was discarded and mRNA was extracted from the protoplast pellet using the Invitrogen Dynabeads mRNA DIRECT Purification kit (61012) according to manufacturer protocol for ‘mini’ extractions. mRNA was eluted with 20 µl of elution buffer and the concentration determined using the Invitrogen Qubit RNA HS Assay kit (Q32852) with the Invitrogen Qubit 4 fluorometer. Concentrations ranged from ~5–9 ng µl^−1^. Library-specific cDNA synthesis and PCR was carried out using the Invitrogen SuperScript IV One-Step RT–PCR System with ezDNase kit (12595100) following manufacturer protocol with minor modifications. All PCR reactions used 30 ng of mRNA template and had a final volume of 50 µl. The forward primer ZmUbi1_5UTR_F3b (5′-GCACACACACACAACCAG-3′) was used with the reverse primer FS_cDNA_R (5′-TGCTAGATCTCGACAGTACG-3′). Cycling conditions were as follows: 55 °C for 10 min (inactivation of ezDNase and first-strand cDNA synthesis), 98 °C for 2 min (inactivation of reverse transcriptase and initial denaturation), 98 °C for 10 s (denaturation), 61 °C for 10 s (annealing), 72 °C for 35 s (extension), 72 °C for 5 min (final extension). Eighteen cycles of denaturation, annealing and extension were performed. The PCR product was column purified using the QIAGEN QIAquick PCR Purification kit (28104) following manufacturer protocol. DNA was eluted with 35 µl of elution buffer and the concentration determined using the Qubit 1X dsDNA HS Assay kit (Q33230) with the Invitrogen Qubit 4 fluorometer. Concentrations ranged from ~8–21 ng µl^−1^.

### Illumina library construction and sequencing

Illumina libraries were generated for each cDNA sample (three replicates of each treatment and control) and the pooled effector library before and after propagation using the Illumina DNA Prep kit (20060059) and IDT for Illumina DNA/RNA UD Indexes Set A (20027213) following manufacturer protocol with minor modifications. Around 120–220 ng of double-stranded (ds)DNA from the cDNA synthesis/PCR or pooled effector library preparation was used as input to achieve on-bead normalization. Right-side library cleanup with purification beads (included in the kit) was carried out following the Illumina protocol, while a 1.8× bead ratio was used for the left-side cleanup of RNA-derived samples to retain smaller amplicons. Illumina library concentrations were measured using the Qubit 1X dsDNA HS Assay kit with the Invitrogen Qubit 4 fluorometer. Concentrations ranged from ~22–29 ng µl^−1^ for RNA-derived samples and ~16–18 ng µl^−1^ for pooled effector library samples. Quality control of library size was carried out using the Agilent TapeStation 2200 with High Sensitivity D1000 ScreenTapes and Reagents (5067-5584 and 5067-5585). Illumina libraries were pooled in equimolar amounts and sequenced on the Illumina NextSeq 500 platform (ACRF Biomolecular Resource Facility, The John Curtin School of Medical Research, Australian National University) using the NextSeq 500/550 Mid Output kit v.2.5 (20024904) and 74 bp paired-end reads. PhiX was spiked in at 5%.

### Differential gene expression analysis

RNA sequencing reads were cleaned using fastp (v.0.22.0)^34^ (--length_required 20) specifying the 5′ untranslated region sequences common to all library transcripts and the Illumina DNA Prep adapter sequence as adapters. The clean reads were aligned to the coding sequences of the 696 cloned effector candidates with HISAT2 (v.2.2.1)^35^ (--very-sensitive; --sp 1,1; --no-spliced-alignment). Mappings where the read pairs map to different transcripts were discarded. Salmon (v.1.8.0)^36^ was used to quantify expression from the HISAT2 alignments. Read counts were imported into DESeq2 (ref. ^37^) with tximport (type = ‘salmon’). Differential expression analysis was performed with DESeq2 (ref. ^37^) and default parameters, followed by lfcShrink (type = ‘apeglm’) to compare each *R* gene treatment with the empty vector control. DESeq2 uses the Wald test to compare expression between two samples and reports *P* values adjusted for multiple testing using the Benjamini and Hochberg method (BH-adjusted *P* values). *P* values of zero (−log_10_ *P*_adj_ = infinity) were converted to the machine-lowest value possible in R (function: .Machine$double.xmin), resulting in a −log_10_ *P*_adj_slightly greater than 300 for those data points (AvrSr50 in both screens; 0100 and the five variants of AvrSr27 in screen 2). Volcano plots were produced with EnhancedVolcano (https://github.com/kevinblighe/EnhancedVolcano). For the mock library screen, *AvrSr50* and *AvrSr27-2* TPM was calculated from the read counts from the HISAT2 alignments and normalized to the *AvrSr35* TPM. Significance was assessed with a two-tailed unpaired *t*-test assuming equal variances. For assessment of pooled effector library propagation, Illumina sequence reads were cleaned, aligned and counted as described above. Construct representation in the pooled effector library before and after propagation was compared on the basis of normalized read counts (Supplementary Fig 7).

### Agroinfiltration of *N. tabacum* and *N. benthamiana* leaves

*N. tabacum* and *N. benthamiana* plants were grown in a growth chamber at 23 °C with a 16 h light period. *Agrobacterium tumefaciens* cultures containing the expression vectors of each construct were grown overnight at 28 °C in LB media with appropriate antibiotic selections (rifampicin/gentamicin/carbenicillin for pAM-GWY vectors and rifampicin/gentamicin/kanamycin for pBIN19-GWY vectors). The cells were pelleted and resuspended in infiltration mix (10 mM MES pH 5.6, 10 mM MgCl_2_, 1,500 μM acetosyringone) to an optical density (OD_600_) of 0.2–0.7 as indicated, followed by incubation at room temperature for 2 h. Cultures were infiltrated into leaves of 4-week-old plants with a 1 ml syringe. For documentation of cell death, leaves were photographed or scanned 2–5 days after infiltration.

### Reporting summary

Further information on research design is available in the Nature Portfolio Reporting Summary linked to this article.

### Supplementary information

Supplementary Information Supplementary Figs. 1–7.

Reporting Summary

Supplementary Table 1 Effector gene candidate library.

## Data Availability

*Pgt* effector expression data used in this study are available in NCBI under BioProject PRJNA516922. Vector sequences and sequence data from library screens are deposited on the CSIRO Data Access Portal at 10.25919/0c6m-mr18 (ref. ^38^).

## Extended data

**Extended Data Table 1:** Plasmids used in this study

Use	Construct	Plasmid name	Plasmid backbone	Cloning method	Source
	AvrSr13	pDONR-AvrSr13	pDONR207	BP-Gateway	-
	AvrSr22	pDONR-AvrSr22	pDONR207	BP-Gateway	-
	AvrSr22b	pDONR-AvrSr22b	pDONR207	synthesis/BP-Gateway	-
	Sr13c	pDONR-Sr13c	pDONR207	synthesis/BP-Gateway	-
	Sr22	pDONR-Sr22	pDONR207	synthesis/BP-Gateway	-
Expression vectors for wheat protoplasts	pUBQ-YFP	pTA22-YFP	pTA22	-	-
	pUBQ- empty vector	pTA22	pTA22	restriction digestion	
	pUBQ gateway cloning vector	pTA22-GW	pTA22	restriction digestion	
	pUBQ gateway cloning vector with PBS	pTA22-GW-PBS	pTA22	restriction digestion	
	pUBQ-RFP	pTA22-RFP	pTA22-GW	LR-Gateway	-
	pUBQ-Sr13c	pTA22-Sr13c	pTA22-GW	LR-Gateway	-
	pUBQ-Sr21	pTA22-Sr21	pTA22-GW	LR-Gateway	-
	pUBQ-Sr22	pTA22-Sr22	pTA22-GW	LR-Gateway	-
	pUBQ-Sr27	pTA22-Sr27	pTA22-GW	LR-Gateway	-
	pUBQ-Sr35	pTA22-Sr35	pTA22-GW	LR-Gateway	-
	pUBQ-Sr50	pTA22-Sr50	pTA22-GW	LR-Gateway	-
	pUBQ-Sr61	pTA22-Sr61	pTA22-GW	LR-Gateway	-
	pUBQ-AvrSr50	pTA22-AvrSr50-PBS	pTA22-GW-PBS	LR-Gateway	-
	pUBQ-AvrSr27-2	pTA22-AvrSr27-2-PBS	pTA22-GW-PBS	LR-Gateway	-
	pUBQ-AvrSr35	pTA22-AvrSr35-PBS	pTA22-GW-PBS	LR-Gateway	-
	pUBQ-AvrSr13	pTA22-0100-PBS	pTA22-GW-PBS	synthesis	
	pUBQ-AvrSr22	pTA22-0336-PBS	pTA22-GW-PBS	synthesis	
	pUBQ-AvrSr2b	pTA22-AvrSr22b-PBS	pTA22-GW-PBS	LR-Gateway	-
	pUBQ-AvrSr2b	pTA22-AvrSr22b-PBS	pTA22-GW-PBS	LR-Gateway	-
Expression vectors in *Nicotiana*	35S-AvrSr50	pBIN19-YFP-AvrSr50	pBIN19-YFP-GTW	LR-Gateway	Chen et al.^13^
	35S-AvrSr27-2	pBIN19-YFP-AvrSr27-2	pBIN19-YFP-GTW	LR-Gateway	Upadhyaya et al.^11^
	35S-Sr13c-YFP	pAM-Sr13c-YFP	pAM-GTW-YFP	LR-Gateway	-
	35S-Sr22-YFP	pAM-Sr22-YFP	pAM-GTW-YFP	LR-Gateway	-
	35S-YFP-AvrSr13	pBIN19-YFP-AvrSr13	pBIN19-YFP-GTW	LR-Gateway	-
	35S-YFP-AvrSr22	pBIN19-YFP-AvrSr22	pBIN19-YFP-GTW	LR-Gateway	-
	35S-YFP-AvrSr22b	pBIN19-YFP-AvrSr22b	pBIN19-YFP-GTW	LR-Gateway	-
	35S-Sr27-YFP	pAM-Sr27-YFP	pAM-GTW-YFP	LR-Gateway	Upadhyaya et al.^11^
	35S-Sr50-YFP	pAM-Sr50-YFP	pAM-GTW-YFP	LR-Gateway	-
