# Supplementary material for: Pooled effector library screening in protoplasts rapidly identifies novel Avr genes
Source: Nat Plants. 2024 Feb 26;10(4):572–80. doi: 10.1038/s41477-024-01641-y (PMC11035141; doi:10.1038/s41477-024-01641-y)
Supplement: Supplementary file 1 — Supplementary Figs. 1–7. [file 41477_2024_1641_MOESM1_ESM.pdf]

nature plants

Letter

https://doi.org/10.1038/s41477-024-01641-y

Pooled effector library screening in protoplasts rapidly identifies novel *Avr* genes

In the format provided by the authors and unedited

Pooled effector library screening in protoplasts rapidly identifies novel *Avr* genes

Taj Arndell[1,2], Jian Chen[1], Jana Sperschneider[1], Narayana M Upadhyaya[1], Cheryl Blundell[1], Nathalie Niesner[1], Megan A. Outram[1], Aihua Wang[1], Steve Swain[1], Ming Luo[1], Michael A Ayliffe[1], Melania Figueroa[1], Thomas Vanhercke[1,]*, Peter N. Dodds[1,]*.

[1] CSIRO Agriculture and Food, GPO Box 1700, Clunies Ross Street, Canberra, Australia, 2601

[2] Current address: Centre for Agriculture and the Bioeconomy, Queensland University of Technology, GPO Box 2434, Brisbane, QLD 4001, Australia

* Authors for correspondence: peter.dodds@csiro.au, thomas.vanhercke@csiro.au

Supplementary Figures

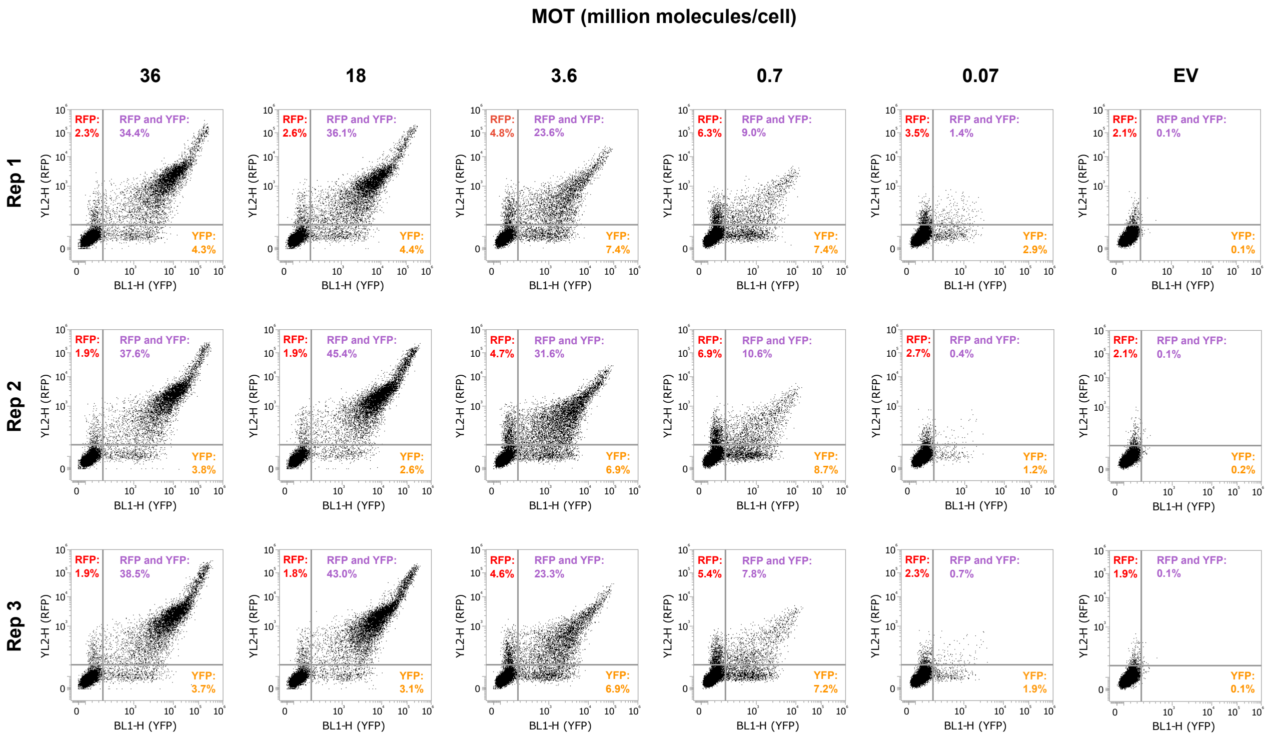

Supplementary Fig. 1. Test for independent transformation of protoplasts using fluorescent reporter-based mock libraries. Wheat protoplasts were transformed with one of a series of mock libraries comprised of *YFP* and *RFP* at various MOTs (million plasmid molecules/cell) along with an empty vector (EV) whose amount was varied such that the combined MOT of the three constructs remained constant at 72 million molecules/cell. Flow cytometry was used to determine the percentage of cells showing fluorescence in the YFP, RFP or both wavelengths, as well as the fluorescence intensity for each cell. Plots show fluorescence levels of each cell (dots) for YFP (X-axis log_{10} scale) and RFP (Y-axis log_{10} scale). Gating parameters for YFP and RFP fluorescence are indicated by the grey lines. Data points in the bottom right (rectangular) quadrant represent cells that express YFP only, data points in the top left (rectangular) quadrant represent cells that express RFP only, and data points in the top right (square) quadrant represent cells that express both YFP and RFP.

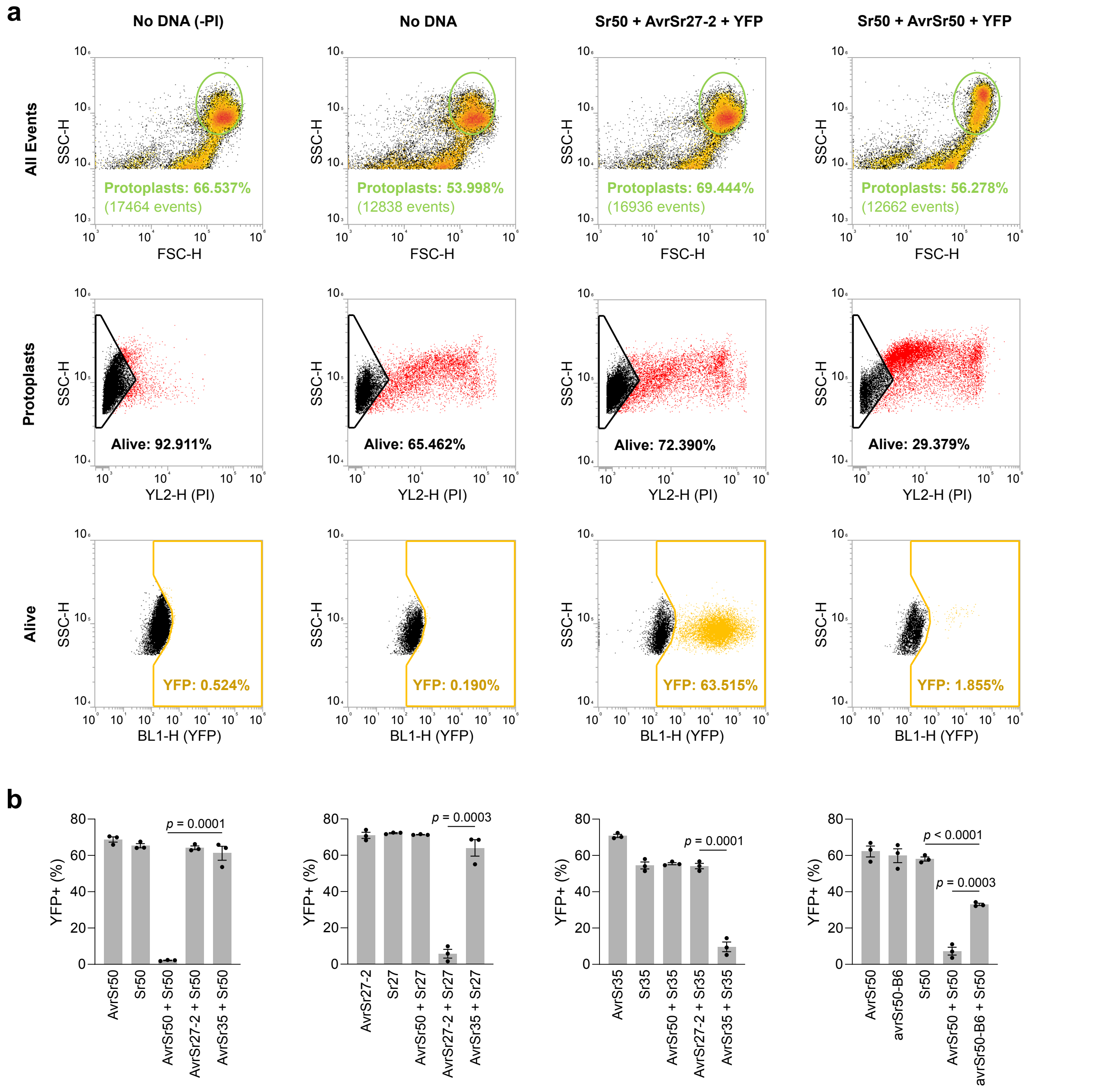

Supplementary Fig. 2. Validation of flow cytometry-based assay for Avr recognition and induced cell death in wheat protoplasts. **a,** Flow cytometry gating. Representative plots show flow cytometry output for wheat protoplasts that were untransformed/unstained (first column), untransformed/stained (second column), and transformed/stained (third and fourth columns). Gates were set to delimit the protoplast population within all events (first row), living cells within the protoplast population (second row), and YFP-positive cells within the living population (third row). Protoplasts were co-transformed with *YFP* and a non-matching *R-Avr* pair (*Sr50* + *AvrSr27-2*) or matching *R-Avr* pair (*Sr50* + *AvrSr50*). Fluorescence levels are plotted on the X-axis for propidium iodide (PI) viability stain (second row) and YFP (third row). A decrease in the percentage of YFP-positive living cells (as seen in the *Sr50* + *AvrSr50* treatment) is indicative of Avr recognition and subsequent cell death. FSC-H, forward scatter; SSC-H, side scatter. **b**, Test for Avr recognition and induced cell death using known *R-Avr* pairs. Wheat protoplasts were co-transformed with *YFP* and either single *Avr* genes (*AvrSr50*, *AvrSr27-2*, *AvrSr35*, or the *avrSr50-B6* virulence allele), single *R* genes (*Sr50*, *Sr27*, or *Sr35*), matching *R-Avr* pairs, or non-matching *R-Avr* pairs as indicated. All gene constructs were delivered at an MOT of 36 million plasmid molecules/cell. The percentage of YFP-positive living cells was determined by flow cytometry. Mean of three replicates with standard error is shown. Significant differences are indicated for relevant pairwise comparisons (two-tailed unpaired *t*-test assuming equal variances).

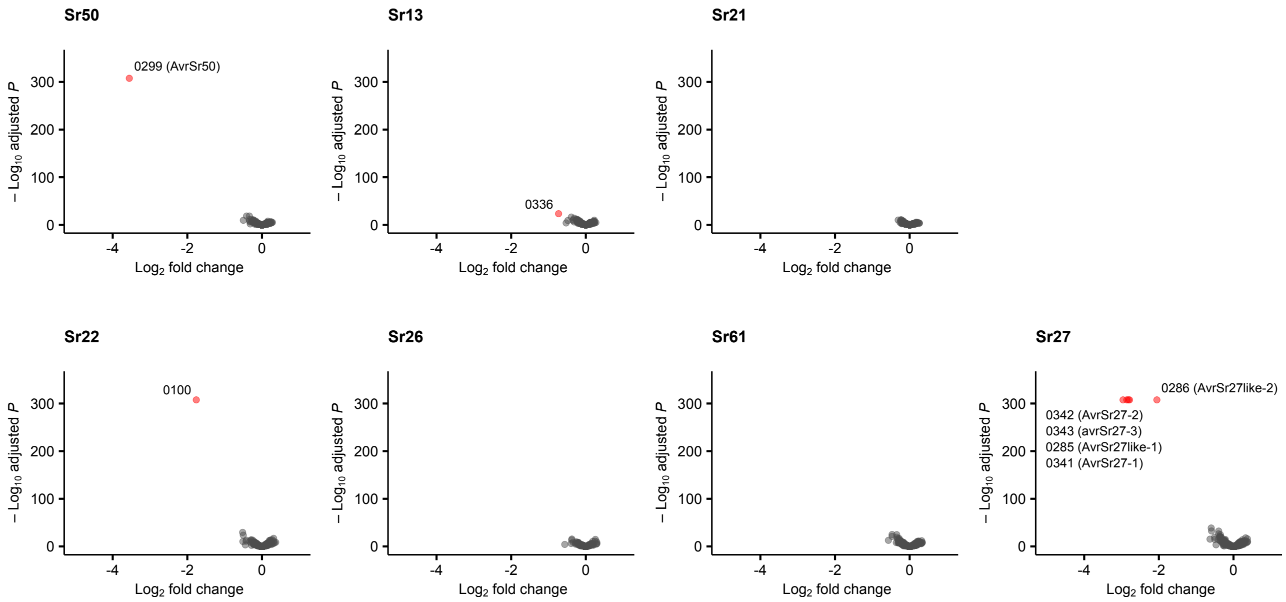

Supplementary Fig. 3. Repeat screening of the pooled stem rust effector library in wheat protoplasts with addition of *Sr27* treatment. Expression analysis of the pooled effector library co-transformed into wheat protoplasts with wheat resistance genes *Sr50*, *Sr27*, *Sr13c*, *Sr21*, *Sr22*, *Sr26* or *Sr61*. Graphs are volcano plots showing differential expression (X-axis) versus adjusted P value (Y-axis) for each of 696 effector constructs (dots). Effectors showing significantly reduced expression in the presence of the *R* gene compared to the empty vector control are labelled with their library ID number. These data are from an independent screen to that presented in Fig. 2d.

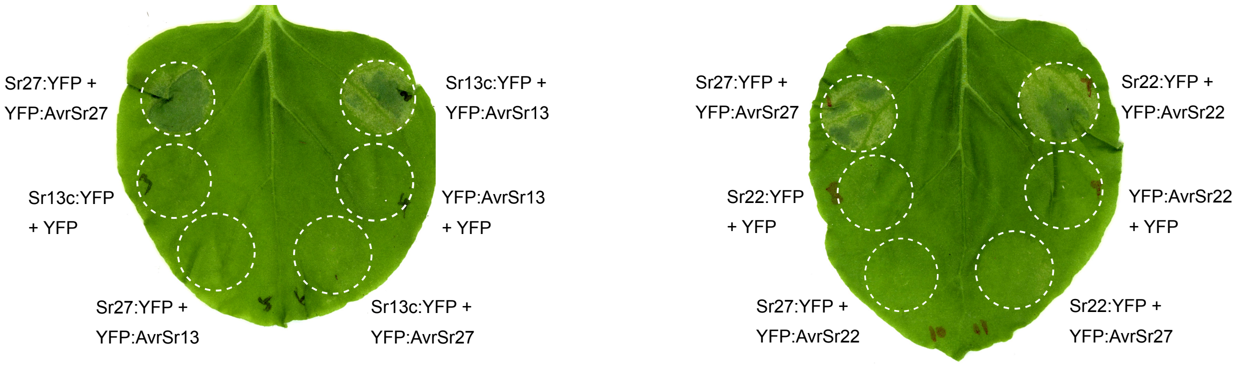

Supplementary Fig. 4. Validation of candidates for *AvrSr13* and *AvrSr22* via agroinfiltration of *N. benthamiana* leaves. Leaf sectors express combinations of *Sr27*, *Sr13c* or *Sr22* C-terminally fused to *YFP* along with *AvrSr13*, *AvrSr22*, or *AvrSr27* N-terminally fused to *YFP*, or with *YFP* alone. Recognition of the Avr protein by the R protein results in cell death, which is visible as discoloured necrotic tissue. Agrobacterial cultures were delivered at OD600 of 0.4 (*R* gene constructs) or 0.7 (*Avr* gene constructs).

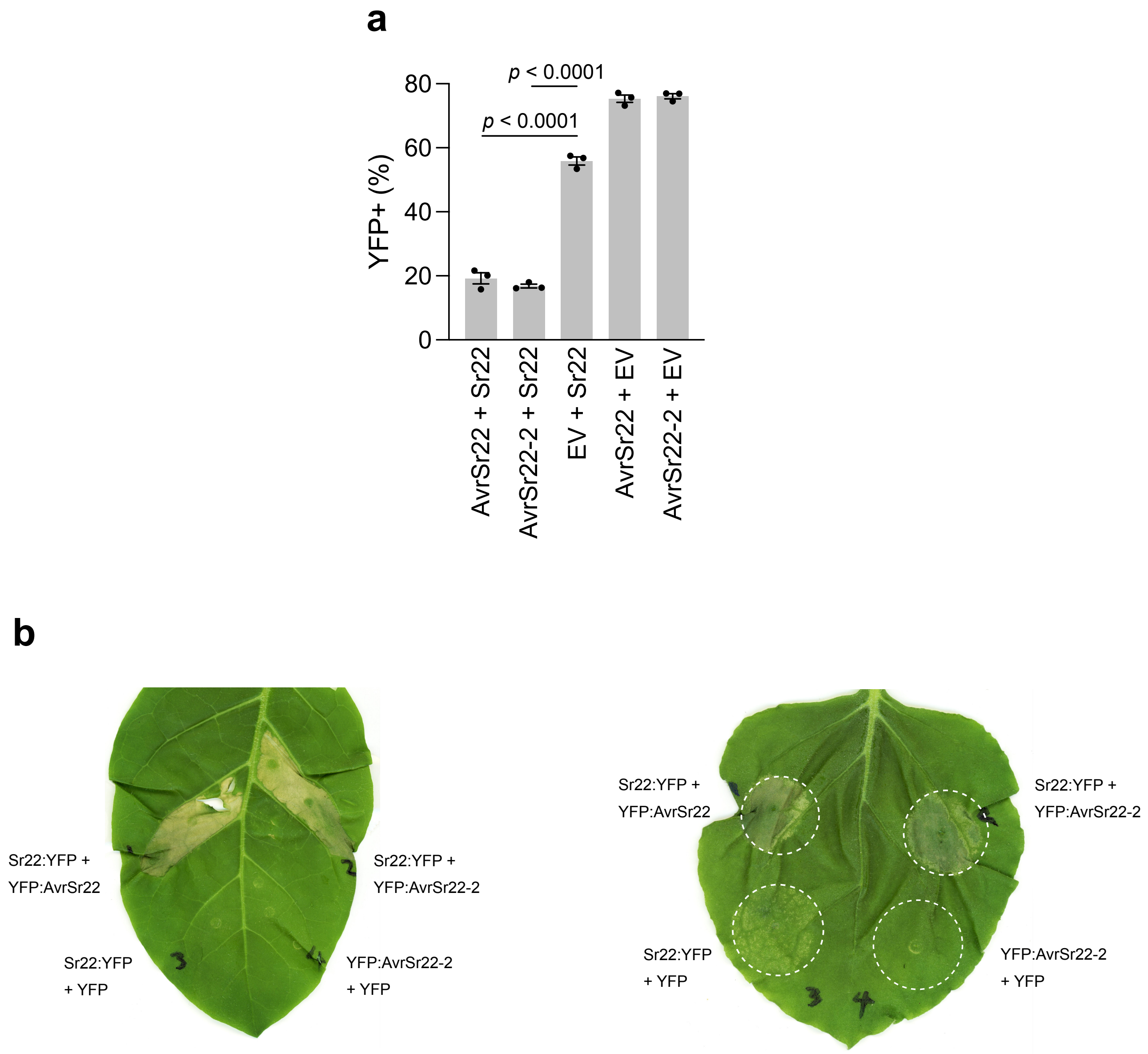

Supplementary Fig. 5. Sr22 recognises both AvrSr22 and AvrSr22-2. **a**, Wheat protoplasts were co-transformed with *YFP*, an *AvrSr22* variant (*AvrSr22* or *AvrSr22-2*) and *Sr22*. For controls, an empty vector (EV) was used as a substitute. All gene constructs were delivered at an MOT of 36 million plasmid molecules/cell. The percentage of YFP-positive living cells was determined by flow cytometry. Mean of three replicates with standard error is shown. Significant differences are indicated for relevant pairwise comparisons (two-tailed unpaired *t*-test assuming equal variances). **b,** Agrobacterium-mediated transient co-expression of *Sr22* (C-terminally fused to *YFP*) with *AvrSr22*, *AvrSr22-2* (N-terminally fused to *YFP*), or *YFP* alone in *N. tabacum* (left) and *N. benthamiana* (right) leaves*.* Agrobacterial cultures were delivered at OD600 of 0.2 (*R* gene constructs) or 0.5 (*Avr* gene constructs). Images were taken five days post-infiltration.

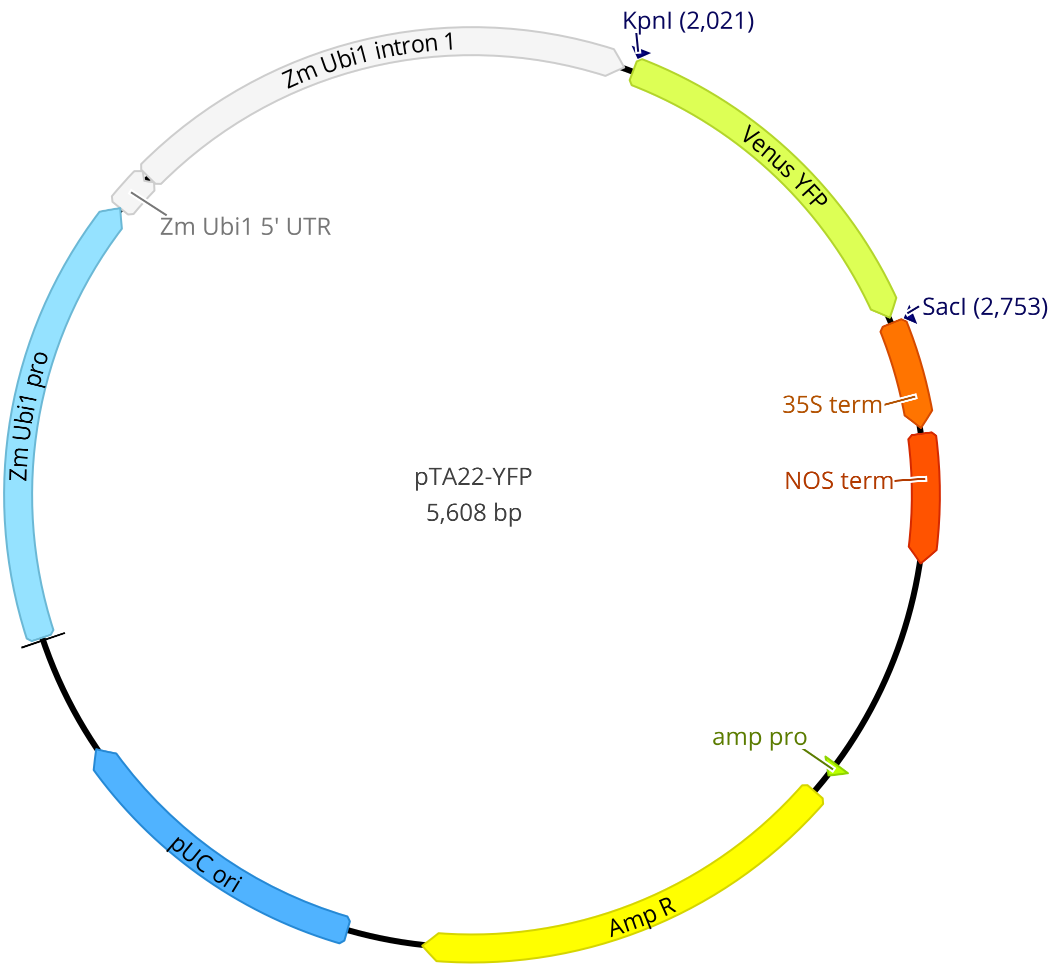

Supplementary Fig. 6. Vector diagram of the YFP reporter construct pTA22-YFP.

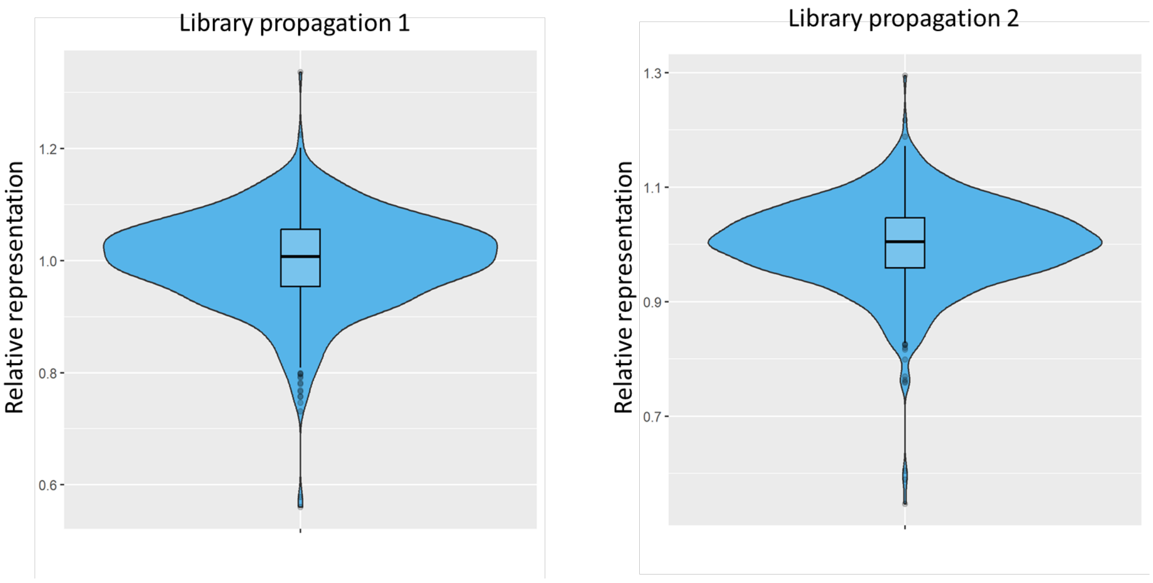

Supplementary Fig. 7. Relative representation of plasmid constructs in effector library after propagation. Constructs were initially pooled in equimolar amounts based on DNA quantification, and then propagated by transformation and re-isolation from *E. coli*. The library preparations before and after propagation were sequenced using Illumina and the representation of each plasmid in the propagated library compared to the original pooled library based on normalised read counts. The violin plot shows the distribution of the relative amounts of each plasmid (n = 696) after propagation compared to the original pool for two library preparations used in the two independent screens in Fig. 2d (left plot) and Supplementary Fig. 3 (right plot). The lower and upper boxplot edges correspond to the first and third quartiles, with the median shown as the middle black line. The upper (lower) whiskers extend from the hinge to the largest (smallest) value that is within 1.5 times the interquartile range of the hinge. Data beyond the end of the whiskers are outliers and shown as dots. The representation of 98% of constructs after propagation was within +/- 20% of that in the original pool.
