# Supplementary material for: Pooled effector library screening in protoplasts rapidly identifies novel Avr genes
Source: Nat Plants. 2024 Feb 26;10(4):572–80. doi: 10.1038/s41477-024-01641-y (PMC11035141; doi:10.1038/s41477-024-01641-y)
Supplement: Supplementary file 2 — Reporting Summary [file 41477_2024_1641_MOESM2_ESM.pdf]

nature portfolio

Corresponding author(s): Peter Dodds, Thomas Vanhercke

Last updated by author(s): Aug 10, 2023

Reporting Summary

Nature Portfolio wishes to improve the reproducibility of the work that we publish. This form provides structure for consistency and transparency in reporting. For further information on Nature Portfolio policies, see our Editorial Policies and the Editorial Policy Checklist.

Statistics

For all statistical analyses, confirm that the following items are present in the figure legend, table legend, main text, or Methods section.

n/a	Confirmed	
☐	☒	The exact sample size (*n*) for each experimental group/condition, given as a discrete number and unit of measurement
☐	☒	A statement on whether measurements were taken from distinct samples or whether the same sample was measured repeatedly
☐	☒	The statistical test(s) used AND whether they are one- or two-sided<br>*Only common tests should be described solely by name; describe more complex techniques in the Methods section.*
☒	☐	A description of all covariates tested
☒	☐	A description of any assumptions or corrections, such as tests of normality and adjustment for multiple comparisons
☐	☒	A full description of the statistical parameters including central tendency (e.g. means) or other basic estimates (e.g. regression coefficient) AND variation (e.g. standard deviation) or associated estimates of uncertainty (e.g. confidence intervals)
☐	☒	For null hypothesis testing, the test statistic (e.g. *F*, *t*, *r*) with confidence intervals, effect sizes, degrees of freedom and *P* value noted<br>*Give P values as exact values whenever suitable.*
☒	☐	For Bayesian analysis, information on the choice of priors and Markov chain Monte Carlo settings
☒	☐	For hierarchical and complex designs, identification of the appropriate level for tests and full reporting of outcomes
☒	☐	Estimates of effect sizes (e.g. Cohen's *d*, Pearson's *r*), indicating how they were calculated

Our web collection on statistics for biologists contains articles on many of the points above.

Software and code

Policy information about availability of computer code

Data collection	no software was used for data collection
Data analysis	RNA sequencing reads were cleaned using fastp 0.22.0. Reads were aligned to reference sequences using HISAT2 2.2.1. Transcript abundances were assessed using Salmon 1.8.0. DESeq2 1.34.0 was used to assess differential expression. Volcano plots were produced with EnhancedVolcano1.12.0. Statistical tests were performed with prism10.0.1. flow cytometry data was analysed using Attune NxT 3.1

For manuscripts utilizing custom algorithms or software that are central to the research but not yet described in published literature, software must be made available to editors and reviewers. We strongly encourage code deposition in a community repository (e.g. GitHub). See the Nature Portfolio guidelines for submitting code & software for further information.

Data

Policy information about availability of data

All manuscripts must include a data availability statement. This statement should provide the following information, where applicable:

- Accession codes, unique identifiers, or web links for publicly available datasets
- A description of any restrictions on data availability
- For clinical datasets or third party data, please ensure that the statement adheres to our policy

Pgt effector expression data used in this study is available in NCBI under BioProject PRJNA516922. Sequence data from library screens is deposited on the CSIRO Data Access Portal under doi: https://doi.org/10.25919/apa3-7v33.

Research involving human participants, their data, or biological material

Policy information about studies with human participants or human data. See also policy information about sex, gender (identity/presentation), and sexual orientation and race, ethnicity and racism.

Reporting on sex and gender	n/a
Reporting on race, ethnicity, or other socially relevant groupings	n/a
Population characteristics	n/a
Recruitment	n/a
Ethics oversight	n/a

Note that full information on the approval of the study protocol must also be provided in the manuscript.

Field-specific reporting

Please select the one below that is the best fit for your research. If you are not sure, read the appropriate sections before making your selection.

☒ Life sciences ☐ Behavioural & social sciences ☐ Ecological, evolutionary & environmental sciences

For a reference copy of the document with all sections, see nature.com/documents/nr-reporting-summary-flat.pdf

Life sciences study design

All studies must disclose on these points even when the disclosure is negative.

Sample size	three biological replicates performed for protoplast transformation experiments experiments as is standard for the field.
Data exclusions	no data were excluded
Replication	Library screening experiments were performed twice with similar results as shown in figure 2 and supp fig 3. Protoplast and nicotiana transformation experiments repeated at least twice with consistent results.
Randomization	not relevant to study as no subjects were allocated to experimental groups.
Blinding	no subjective scoring requiring blinding were performed. Automated quantification of samples.

Reporting for specific materials, systems and methods

We require information from authors about some types of materials, experimental systems and methods used in many studies. Here, indicate whether each material, system or method listed is relevant to your study. If you are not sure if a list item applies to your research, read the appropriate section before selecting a response.

Materials & experimental systems

n/a	Involved in the study	
☒	☐	Antibodies
☒	☐	Eukaryotic cell lines
☒	☐	Palaeontology and archaeology
☒	☐	Animals and other organisms
☒	☐	Clinical data
☒	☐	Dual use research of concern
☒	☐	Plants

Methods

n/a	Involved in the study	
☒	☐	ChIP-seq
☐	☒	Flow cytometry
☒	☐	MRI-based neuroimaging

Flow Cytometry

Plots

Confirm that:

☒ The axis labels state the marker and fluorochrome used (e.g. CD4-FITC).

☒ The axis scales are clearly visible. Include numbers along axes only for bottom left plot of group (a 'group' is an analysis of identical markers).

☒ All plots are contour plots with outliers or pseudocolor plots.

☒ A numerical value for number of cells or percentage (with statistics) is provided.

Methodology

Sample preparation	Wheat (Triticum aestivum) seeds were planted in 13 cm pots (12 seeds per pot) containing Martins Seed Raising and Cutting Mix supplemented with 3 g/L osmocote. Seedlings were grown in a growth cabinet at 24°C on a cycle of 12 hours light (~100 µmol m-2 s-1) and 12 h dark, for 7–8 days. Wheat cultivar Fielder was used unless otherwise stated. Protoplast isolation and transformation was carried out as described in Arndell, et al 2019 BMC Biotechnology 19, 71, except that released protoplasts were filtered through a 40µm nylon cell strainer and final suspension was in 6 mL MMG solution (4 mM MES-KOH (pH 5.7), 0.4 M mannitol, 15 mM MgCl2).
Instrument	Invitrogen Attune NxT Flow Cytometer
Software	Attune NxT Software 3.1
Cell population abundance	No post-sort fractions were collected through flow cytometry
Gating strategy	The first gate (FSC / SSC) selected the protoplast population. The second gate was set with reference to an unstained and untransformed control to select the living sub-population of protoplasts (negative for propidium iodide staining) in stained samples. The third gate was set with reference to a propidium iodide-stained and untransformed control to determine the percentage of living cells that were YFP-positive in transformed samples.

☒ Tick this box to confirm that a figure exemplifying the gating strategy is provided in the Supplementary Information.
